# Potential analgesic effects of a novel *N*-acylethanolamine acid amidase inhibitor F96 through PPAR-α

**DOI:** 10.1038/srep13565

**Published:** 2015-08-27

**Authors:** Longhe Yang, Long Li, Ling Chen, Yanting Li, Huixia Chen, Yuhang Li, Guangnian Ji, Donghai Lin, Zuguo Liu, Yan Qiu

**Affiliations:** 1 Medical College, Xiamen University, Xiamen, Fujian 361005, P. R. China; 2 Engineering Research Center of Marine Biological Resource Comprehensive Utilization, Third Institute of Oceanography , State Oceanic Administration, Xiamen, Fujian 361005, P. R. China; 3 Department of Chemistry and The Key Laboratory for Chemical Biology of Fujian Province, College of Chemistry and Chemical Engineering, Xiamen University, Xiamen, Fujian 361005, P. R. China

## Abstract

Pharmacological blockade of *N*-acylethanolamine acid amidase (NAAA) activity is an available approach for inflammation and pain control through restoring the ability of endogenous PEA. But the recently reported NAAA inhibitors suffer from the chemical and biological unstable properties, which restrict functions of NAAA inhibition *in vivo*. It is still unrevealed whether systematic inhibition of NAAA could modulate PEA-mediated pain signalings. Here we reported an oxazolidinone imide compound 3-(6-phenylhexanoyl) oxazolidin-2-one (F96), which potently and selectively inhibited NAAA activity (IC_50_ = 270 nM). Intraperitoneal (i.p.) injection of F96 (3–30 mg/kg) dose-dependently reduced ear edema and restored PEA levels of ear tissues in 12-*O*-Tetradecanoylphorbol-13-acetate (TPA) induced ear edema models. Furthermore, F96 inhibited acetic acid-induced writhing and increased spared nerve injury induced tactile allodynia thresholds in a dose-dependent manner. Pharmacological effects of F96 (10 mg/kg, i.p.) on various animal models were abolished in PPAR-α^−/−^ mice, and were prevented by PPAR-α antagonist MK886 but not by canabinoid receptor type 1 (CB_1_) antagonist Rimonabant nor canabinoid receptor type 2 (CB_2_) antagonist SR144528. Zebrafish embryos experiments showed better security and lower toxicity for F96 than ibuprofen. These results revealed that F96 might be useful in treating inflammatory and neuropathic pain by NAAA inhibition depending on PPAR-α receptors.

Endogenous fatty acid ethanolamides (FAEs) play important roles in modulation of a wide range of physiological and pathological processes, especially for inflammatory and neuropathic pain^1^,^2^. Increasing FAEs as anandamide (AEA), oleoylethanolamide (OEA) and palmitoylethanolamide (PEA) *in vivo* effectively elicit anti-inflammation and anti-nociception in various animal models through ‘Nature’s own mechanisms’^3^,^4^,^5^. These lipid messengers are supposed to be produced and degraded by enzymes in mammalian cells to maintain cellular homeostasis, which are different with the classic neurotransmitters stored in vesicles at all time^6^. Among them, PEA is approved as ‘Dietary Foods for Special Medical Purposes’ to treat chronic pain, which is commercially available in Italy and several other European countries. PEA is also widely studied in recent clinical trials, such as temporomandibular joint inflammatory pain^7^ and various chronic pain^8^,^9^, but its analgesic effects is far from powerful^10^.

It is revealed that PEA is biosynthesized from *N*-palmitoyl-phosphatidyl-ethanolamine to exert analgesic and anti-inflammatory effects, which are mainly attributed to activation of nuclear receptor peroxisome proliferator-activated receptor-α (PPAR-*α*)^6^,^11^. While, PEA can also be observed to be reduced obviously during pathological conditions as in rat granuloma tissue^12^, carrageenin induced mice paw edema skin^13^, and sciatic nerve tissue after chronic constriction injury (CCI)^14^. PEA is degraded and deactivated preferentially by a lysosomal enzyme NAAA to palmitic acid and ethanolamine^15^,^16^. Inhibition of NAAA might be an available drug target for inflammation and pain control by modulating the tissue levels of PEA^17^.

Several types of NAAA inhibitors have been identified so far^18^,^19^,^20^,^21^,^22^,^23^,^24^,^25^, among which *β*-lactones were comprehensively discovered to shed light on the endogenous PEA mediated signaling on inflammation and pain^14^,^23^. *N*-[(3*S*)-2-oxo-3-oxetanyl]-3-phenylpropanamide [(*S*)-OOPP] inhibited rat NAAA with a median effective concentration (IC_50 _= 0.42 μM) by normalizing PEA levels in activated inflammatory cells, and reducing tissue reactions to spinal cord trauma with local administration^23^. Recently, a more potent anologue of (*S*)-OOPP, 5-phenylpentyl *N*-[(2S,3R)-2-methyl-4-oxo-oxetan-3-yl] carbamate (ARN077), inhibited human NAAA with IC_50_ of approximate 7 nM, normalized FAE levels in inflamed mouse ear skin, and attenuated the hyperalgesia and allodynia in mice which was evoked by carrageenan and sciatic nerve injury with local administration^14^. These compounds contain an unstable *β*-lactone moiety which is essential for NAAA inhibition, thereby the labile structure hinders their potential application of systemic administration^17^. In 2014, a series of 3-aminoazetidin-2-ones which the *β*-lactone ring was replaced by *β*-lactam was reported by Fiasella, A. *et al.* as a new class of plasma stable NAAA inhibitors , and exerted systemically activity^26^. In another paper, AM9053 was reported as the first systemically active NAAA inhibitor to increase PEA levels in the colon of mouse models of IBD^27^. But the therapeutic potential and the related mechanism of systemically NAAA inhibition in controlling pain still needs more pharmacological tools and detailed revealing.

Previously, we reported a series of acyl pyrrolidine compounds, such as 1-(2-Biphenyl-4-yl)ethyl-carbonyl pyrrolidine, exhibited good biological stability and inhibited rat NAAA with IC_50_ of 2.12 μM and normalized PEA level accompanied with reduced iNOS and IL-6 inflammatory mediators mRNA express in LPS induced RAW264.7 mice macrophage cells^21^. Herein, we disclose a highly potent and stable NAAA inhibitor, 3-(6-phenylhexanoyl)oxazolidin-2-one (F96), which is suitable for systemic administration. In this report, we described the pharmacological profiles of F96, and its underlying mechanism on inflammatory and neuropathic pain after systemic NAAA inhibition.

## Results

### F96 is a selective and stable NAAA inhibitor

**Figure 1:**
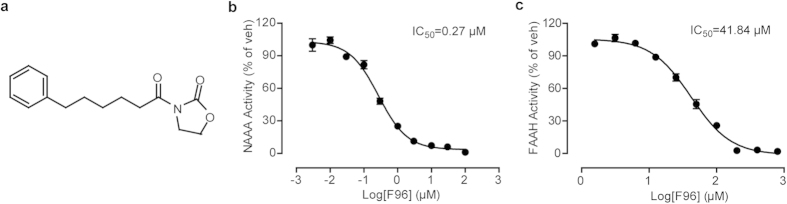
Characterization of the NAAA inhibitor F96. (**a**) Structure of compound F96. (**b**) Concentration-dependent inhibition of extracted recombinant rat NAAA (rNAAA) activity by F96. (**c**) Concentration-dependent inhibition of extracted recombinant rat FAAH (rFAAH) activity by F96.

Structural modification based on oxazolidinone imides led us to identify 3-(6-phenylhexanoyl) oxazolidin-2-one (compound F96, Fig. 1a) with a potent NAAA inhibitory activity (IC_50_ for rat NAAA: 269.3 ± 22.4 nM, Fig. 1b, for human NAAA: 268.6 ± 43.8 nM). Incubation of F96 in various concentrations (10 nM-100 μM) in intact HEK-293-rNAAA cells revealed that the IC_50_ of F96 in cells was 419.2 ± 39.6 nM. In addition, F96 exhibited 150-fold selectivity for NAAA over FAAH (IC_50_ for rat FAAH: 42.05 ± 1.92 μM, Fig. 1c) and did not show enough inhibitory activity for MAGL and acid ceramidase (AC) in concentration of 10 μM *in vitro* (Table 1).

Considering about whether F96 could directly act as a PPAR-*α* agonist, we engineered 293T cells to express a luciferase reporter gene together with the ligand-binding domain (LBD) of human PPAR-α fused to the yeast GAL4 DNA-binding domain. In transactivation assay, F96 had no effect on PPAR-α compared with DMSO in all concentrations (Fig. S1a).

We also conducted the PPAR-α competitive binding assay (LanthaScreen^®^ TR-FRET PPAR-α competitive binding assay kit, Life Technologies™) to examine that if F96 could bind to PPAR-α. The results suggested that F96 did not bind to the LBD of PPAR-α even in high doses of 10 μM (Fig. S1b). Taken together, F96 is a selective NAAA inhibitor and do not directly active PPAR-α through binding it.

We further evaluated the stability of F96 in various chemical and biological conditions. Results indicated that this compound has excellent stability in either acidic medium (pH 5.0: t_1/2 _> 1440 min) or basic medium (pH 7.4: t_1/2 _> 1440 min), which also revealed a considerable metabolic rate when incubated with 80% rat plasma under 37 °C physiological conditions (*t*_1/2 _= 188 ± 17 min).

### Effects of F96 on TPA induced ear edema

**Figure 2:**
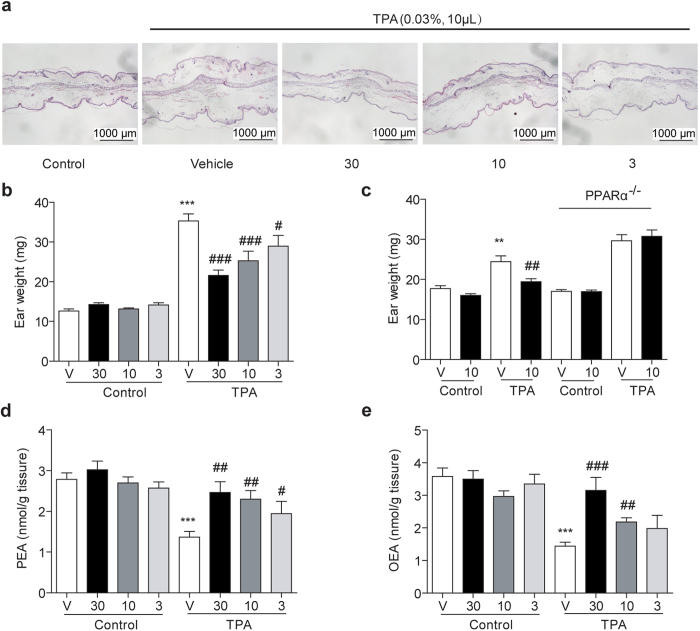
Effects of F96 on TPA induced ear edema and ear tissues levels of fatty acid ethanolamides. (**a**) H&E-stained sections of normal mouse ear (Control), TPA-induced irritant dermatitis (Vehicle) and various doses (30, 10, 3 mg/kg) of F96 treated. (**b**) F96 (30, 10, 3 mg/kg) dose-dependently reduced ear weight. (**c**) F96 (10 mg/kg) reduced ear weigh in wild type 129 s mice but not in PPAR-α knockout mice. (**d–e**) F96 normalized PEA and OEA levels in a dose-dependent manner after TPA application for 3 h in C57BL/6J mice. **p < 0.01 and ***p < 0.001 *vs* Control vehicle-treated group. #*p *< 0.05, ##*p *< 0.01 and ###*p *< 0.001 *vs* TPA+F96-treated group. *n *= 8–10.

TPA induced mice ear edema model is always applied to evaluate the anti-inflammatory effects of lead compounds. Here, we investigated the pharmacological effects of F96 in this model. In consistent with previously reports^4^, TPA (0.03% in acetone, 10 μL) was smeared onto left ears of mice to cause local edema to compare with that of the right vehicle ones (acetone, 10 μL). Swelling condition was revealed by H&E assay (Fig. 2a) and ear tissue weight (Fig. 2b; TPA, 35.31 ± 1.78 g *vs* vehicle, 12.66 ± 0.52 g; *P *< 0.001). Simultaneous administration of F96 (3–30 mg/kg, i.p.) for 3 hr reduced ear edema and ear weight (Fig. 2a,b) in a dose dependent manner, and partially normalized PEA and OEA tissue levels (Fig. 2d,e). Moreover, the anti-edema effects of F96 were completely lost in PPAR-α null mice (Fig. 2c). These data suggested that F96 reduced PEA degradation in pathological conditions by inhibiting NAAA, which elevated the endogenous FAEs to activate PPAR-α receptor to exert anti-edema effects on TPA induced mice ear inflammation.

### Effects of F96 in acetic acid induced visceral pain

**Figure 3:**
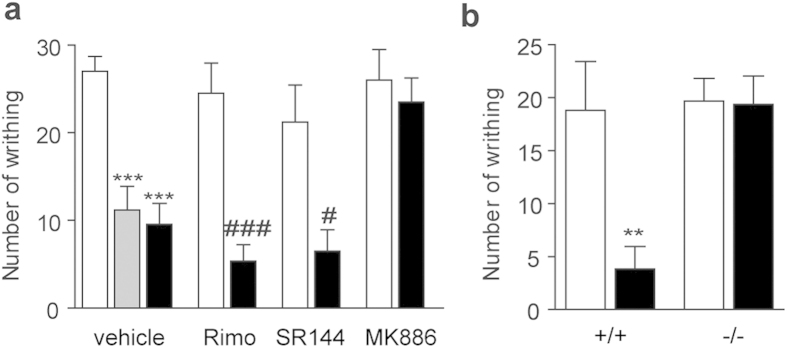
F96 suppressed pain responses elicited by intraperitoneal injections of acetic acid in mice. (**a**) Number of writhing (assessed episodes in 20 min after acetic acid injection) reduced after indomethacin (10 mg/kg, i.p., Gray bars) and F96 (10 mg/kg, i.p., closed bars) administration. PPAR-α antagonist MK886 (2 mg/kg, i.p.) prevented the anti-nociceptive effects of F96. CB_1_ antagonist Rimonabant (1 mg/kg, i.p.) and CB_2_ antagonist SR144528 (1 mg/kg, i.p.) did not abolish the analgesic effects of F96. (**b**) Effects of vehicle (white bars) or F96 (10 mg/kg, i.p., black bars) on acetic acid induced writhing in wild-type 129 s mice (+/+) and PPAR-α knockout mice (−/−).

To evaluate whether systemic administration of NAAA inhibitors attenuated behavioral responses to noxious stimuli in rodents, we examined the effects of F96 on nociceptive responses evoked by acetic acid injection. Acetic acid induced writhing was reversed by F96 (10 mg/kg, i.p.) (Fig. 3a), which were similar with the analgesic effects of cyclooxyenase inhibitor indomethacin at the same dose (10 mg/kg, i.p.) (Fig. 3a). Previous reports indicated that PEA might increase the activity of endogenous agonists of CB receptors *via* ‘entourage effects’^28^, which we did not completely detect. So, we designed additional experiments to reveal whether CB_1_ or CB_2_ was involved in anti-writhing mechanism of F96. As showed in Fig. 3a, the anti-nociceptive effects of F96 (10 mg/kg; i.p.) were not blocked by the selective CB_1_ antagonist Rimonabant (1 mg/kg; i.p.) or by CB_2_ antagonist SR144528 (1 mg/kg; i.p.), but was blocked by PPAR-α antagonist MK886 (2 mg/kg; i.p.). We further employed PPAR-α^−/−^ mice as a complementary genetic model to confirm the role of PPAR-α in mediating the analgesia of F96. As showed in Fig. 3b, genetic disruption of PPAR-α prevented the nociceptive adaptations caused by NAAA inhibition totally. These findings indicated that pharmacological blockade of NAAA systemically could inhibit acetic acid-induced nociceptive responses through PPAR-α receptor rather than cannabinoid receptors.

### Effects of F96 on SNI induced neuropathic pain

**Figure 4:**
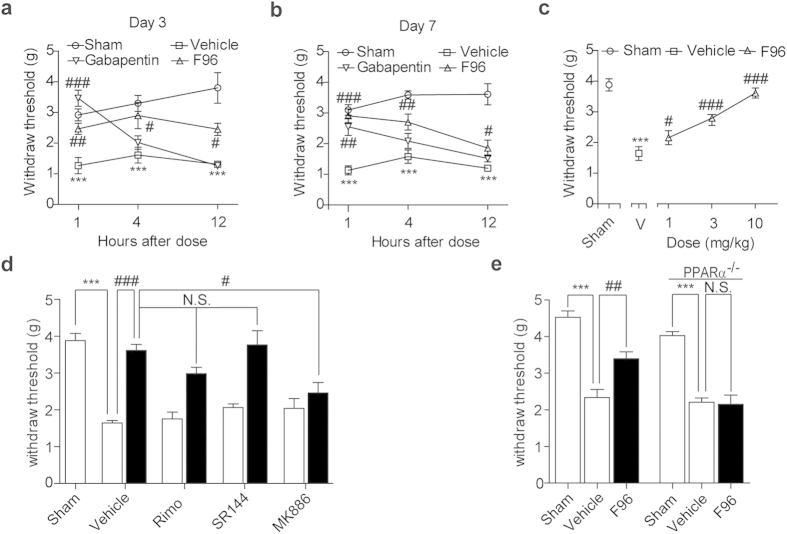
Analgesic effects of F96 in the SNI models. Time-course effect of vehicle (5% PEG/5% Tween-80 in saline, 10 mL/kg, i.p., open squares), F96 (10 mg/kg, i.p., open triangle) and gabapentin (40 mg/kg, i.p., open inverted triangle) on mechanical allodynia in SNI mice at (**a**) 3^rd^ day post-surgery, and (**b**) the 7^th^ day post-surgery. Mechanical allodynia were measured 1, 4 and 12 h after treatment. Sham, sham-operated mice (open cycles). (**c**) Dose-response of F96 (open triangles) on mechanical allodynia in SNI mice 7^th^ day post surgery. Withdraw threshold was assessed 1 h after F96 treatment. Sham, sham-operated mice (open cycle). (**d**) PPAR-α antagonist MK886 (2 mg/kg, i.p.) prevented the anti-allodynia effects of F96 (10 mg/kg, i.p., closed bars). CB_1_ antagonist Rimonabant (1 mg/kg, i.p.) and CB_2_ antagonist SR144528 (1 mg/kg, i.p.) were ineffective. (**e**) F96 (10 mg/kg, i.p.) increased mechanical paw withdraw threshold in wild-type 129 s mice, but not in PPAR-α knockout mice. Results are expressed as mean ± SEM (*n *= 6–10 each group).

We then evaluated the ability of systematic NAAA inhibition to alleviate persistent pain by sciatic nerve injury (SNI). C57BL/6J mice were treated with vehicle, F96 (10 mg/kg, i.p.), gabapentin (30 mg/kg, i.p.) on the 3^rd^ day and the 7^th^ day after surgery, respectively. Sham surgery was conducted as control group. The withdraw thresholds were investigated at 1 h, 4 h and 12 h after each drug administration. As shown in Fig. 4a,b, SNI induced significant decrease of mechanical withdraw thresholds comparing with sham-operated group, which were detected by allodynia test. NAAA inhibition increased pain withdraw thresholds responding to mechanical stimuli on both the 3^rd^ and 7^th^ day post-surgery (Fig. 4a,b). Compound F96 indicated significant analgesic effects at 4 h after drug administration and the pharmacological responses wearing off with time, although which lasted up to 12 h. In contrast, experimental data indicated that the positive control drug gabapentin was only effective on the first hour after treated. What’s more, F96 (1, 3, 10 mg/kg) produced dose-dependent attenuation of mechanical hypersensitivity on the 7^th^ day post-surgery (Fig. 4c). The antinociceptive effects of F96 (10 mg/kg, i.p.) could be blocked by the selective PPAR-α antagonist MK886 (2 mg/kg, i.p.) (Fig. 4d), but not CB_1_ antagonist Rimonabant (1 mg/kg; i.p.) nor CB_2_ antagonist SR144528 (1 mg/kg; i.p.). Genetic model experiments revealed that analgesic effects of NAAA inhibition were invalid in PPAR-α^−/−^ mice when compared with that of the wild-type groups (Fig. 4e).

### Preliminary toxicity of F96 on zebrafish

**Figure 5:**
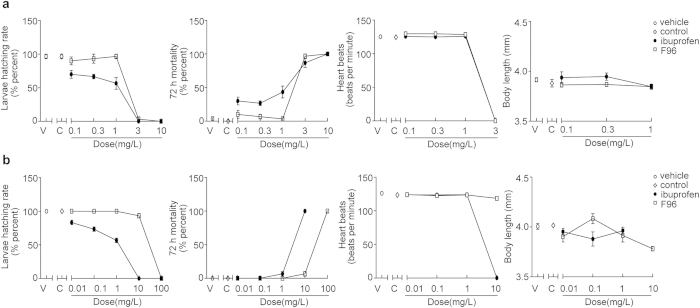
Primary toxicity evaluation of F96 in the zebrafish models. The larvae hatching rate, total mortality, average heartbeats, and body length of the embryos undergoing (**a**) 2/4 celled stages and (**b**) 24 h post-fertilization and exposing to different solutions on 72 h. Vehicle, zebrafish medium (open cycles). Control, zebrafish medium containing 0.1% DMSO (open rhombus). Ibuprofen, zebrafish medium containing different concentrations of ibuprofen (black cycles). F96, zebrafish medium containing different concentrations of ibuprofen (open square).

Two experiments were performed to preliminarily evaluate the toxicity of F96 by comparing with a commercial nonsteroidal antiinflammatory drug (NSAID) ibuprofen in zebrafish models. The total mortality, hatching number, body length and heartbeats of zebrafish embryos treated with various concentrations of F96 and ibuprofen are shown in Fig. 5. In experiment one, the embryos undergoing early cleavage (two/four celled stages) were exposed to the compounds assessed for 72 h. About half of the embryos died when treated with 1 mg/L ibuprofen, while almost all embryos survived with the same dose of F96 (Fig. 5a). The survival rate of embryos incubated in 0.3 mg/L and 0.1 mg/L of F96 were also much higher than ibuprofen. Other parameters such as body length and heartbeats remained nearly the same to controls.

In experiment two, the embryos undergoing 24 h post-fertilization were exposed to the compounds assessed for 72 h. None of the embryos survived in 10 mg/L ibuprofen solution, while all the embryos survived in F96 groups of the same dose (Fig. 5b). In the 0.01 mg/L, 0.1 mg/L and 1 mg/L ibuprofen exposure groups, there was a decrease on the number of hatched larvae. Instead, all the embryos of F96 group hatched in these doses. There was no obvious change in other parameters comparing to control groups. These data suggested that F96 exhibited less toxicity and more safety than ibuprofen.

### Open field assay of F96

**Figure 6:**
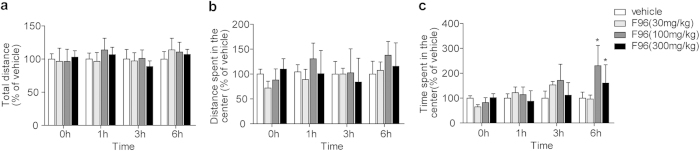
Open field tests. F96 (30, 100, 300 mg/kg, i.p.) did not affect the total distance (**a**), center distance moved in the arena (**b**) all the time. High dose of F96 (100, 300 mg/kg, i.p.) slightly increased the time spent in the center (**c**) only on 6 h after administration. *p < 0.05, compared with vehicle group.

Behavioral studies of mice with F96 treatment in an open field assay revealed that no significant alteration in locomotion was observed even in high dose drug administration. There was no obvious difference between F96 (30, 100, 300 mg/kg, i.p.) and vehicle regarding the total distance, center distance moved in the arena (Fig. 6a,b) for 6 h. High dose administration of F96 (100, 300 mg/kg, i.p.) caused slightly alteration for the time spent in the center area (Fig. 6c) only after treated for 6 h.

## Discussion

It is revealed that activation of CB_1_ receptor may cause episodes of psychosis and panic in some experiments^29^. Dual FAAH/MAGL inhibition caused potent analgesic effects and also led to hypomotility and catalepsy^30^. NAAA inhibition is supposed to preferentially restoring endogenous PEA to engage PPAR-α receptors rather than modulating CB receptors to avoid the potential CNS side effects, which was still unexplored *in vivo* for the absence of NAAA knockout mice and stable inhibitors. Here we propose a biological and chemical stable oxazolidinone compound F96 as a novel pharmacological tool to evaluate the detailed functions and mechanisms of systematic NAAA inhibition in inflammatory and neuropathic pain.

In the TPA induced mice ear edema experiment, decreased levels of PEA and OEA were observed in inflammatory conditions, which could be recovered to normal levels by NAAA inhibition with dose dependence. Meanwhile, ear tissues levels of the most important endocannabinoid AEA was not modulated by F96 (Fig. S2). Thus, the first main result of our research is that selective NAAA inhibition systematically restoring PEA and OEA rather than AEA in the inflammatory tissues. F96 could not exert anti-inflammatory and anti-nociceptive effects in PPAR-α knockout mice, and the pharmacological effects of F96 could also be blocked by MK886, a PPAR-α antagonist. These data confirmed that the pharmacological response of NAAA inhibition should directly depend upon the activation of PPAR-α.

Previous reports showed that inhibition of FAAH activity by URB937 elevated both substrates of AEA and PEA to cause marked analgesic responses in rodent models of acute and persistent pain^31^. The pharmacological effects of URB937 could be prevented by both the CB_1_ antagonist (Rimonabant and AM251) and the PPAR-α antagonist MK886, which was interpreted as two pathways were both involved in the action of FAAH inhibition^31^. To date, it is widely recognized that PEA, as well as classical endocannabinoids, plays a critical role in pain modulation^1^. But the role of AEA and PEA in alleviating inflammatory and neuropathic pain respectively was not well distinguished. Ben-shabat *et al.* described the ‘entourage effect’ of PEA for the first time in 1998, which meant that PEA might act indirectly by increasing the activity or reducing the degradation of endogenous agonists of CB receptors^32^. But in the present study, we showed that CB antagonists, Rimonabant and SR144528, could not reverse the analgesic response of F96, which further identified that systematic NAAA inhibition did not alter the expression of AEA and 2-AG, and even did not engage the CB receptors. These findings suggested the second important results that the elevation of PEA alone *via* inhibition of NAAA could elicit analgesic functions without the alteration of AEA. An open field assay also confirmed that the potential psychoactive response of F96 in locomotion was not observed even in high doses drug treatment. Thus, systematic administration of NAAA inhibitors would theoretically be devoid of unwanted central effects induced by steady elevation of AEA and sustained activation of CB receptors.

Growing evidence supports the communication between prostaglandins pathway and endocannabinoids pathway in inflammatory and neuropathic pain^33^. Inhibition of COX-2 elevates the tissue levels of PEA and AEA, in contrast, overexpression of these lipids down-regulates COX-2 levels and activities^34^. Inhibitors of COX-1 and COX-2 exhibited extensive anti-inflammation and anti-nociceptive effects, but they were always at risk of gastrointestinal hemorrhaging and cardiovascular events. In this study, we compared the NAAA inhibitor with a commercial OTC drug ibuprofen in zebrafish embryos tests. The experimental data suggested that F96 exhibited less toxicity and more safety than ibuprofen, which provided a premise for developing a new candidate in anti-inflammatory and analgesic research field.

## Methods

### Drugs and reagents

MK886, SR144528, Rimonabant were purchased from Cayman Chemical (Michigan, USA). Gabapentin and indomethacin were purchased from Sigma-Aldrich (Shanghai, China). All durgs were dissolved in vehicle (5% Tween 80, 5% PEG 400 in saline solution) with various concentrations as indicated.

### Synthesis of F96

A solution of the 6-phenylhexanoic acid (0.55 mmol) and dimethylformamide (0.05 mL) in CH_2_Cl_2_ was added with oxalyl dichloride (0.66 mmol) under nitrogen at 0 °C. The reaction mixture was then stirred at 0 °C under reduced pressure. Then 2-oxazolidone (0.5 mmol) in THF was added slowly in the presence of n–BuLi (0.55 mmol, 0.2 mL hexane) under nitrogen at −78 °C. After being stirred at −78 °C for 10 min, a solution of acid chloride (0.55 mmol) in THF was slowly added. The reaction mixture was stirred at −78 °C for 0.5 h, and allowed to warm slowly to room temperature in 5 h. The reaction was quenched with a saturated NH_4_Cl solution and extracted with EtOAc. The combined organic layers were dried over anhydrous Na_2_SO_4_, filtered and concentrated under reduced pressure. The residue was purified by flash chromatography on silica gel to afford compound F96 as white crystals. ^1^H NMR (400 MHz, CDCl_3_) *δ* 1.36–1.44 (m, 2 H), 1.61–1.73 (m, 4 H), 2.61 (t, *J *= 7.6 Hz, 2 H), 2.90 (t, *J *= 7.6 Hz, 2 H), 3.97 (t, *J *= 8.4 Hz, 2 H), 4.36 (t, *J *= 8.4 Hz, 2 H), 7.16–7.18 (m, 3 H), 7.24–7.28 (m, 2 H) ppm; ^13^C NMR (100 MHz, CDCl_3_) *δ* 24.0, 28.6, 31.1, 34.9, 35.6, 42.4, 61.9, 125.5, 128.2, 128.3, 142.4, 153.4, 173.3 ppm.

### Ethics statement

All experiments were carried out in accordance with ‘Guide and Care and Use of Laboratory Animals’ from Narional Institutes of Health (NIH) and approved by the Animal Care and Use Committees of Xiamen University in China.

### Cells and animals

HEK293 and RAW264.7 cells were bought from American Type Culture Collection (ATCC, Beijing, China) and were cultured in Dulbecco’s Modified Eagle Medium (DMEM, Hyclone, Beijing, China) supplemented with 10% FBS (Gibco^®^, Shanghai, China). ICR mice, Kunming mice and C57BL/6J mice were obtained from Shanghai Laboratory Animal Center (Shanghai, China). 129 s mice (+/+) and PPAR-α knockout mice (−/−) were from Jackson Laboratory (Bar Harbor, ME, USA). Zebrafish embryos were kind gifts from Prof. Wanshu Hong (School of Life Science, Xiamen University).

### Enzyme activity assay

The inhibition effects of F96 on enzymes were assessed by enzyme activity assay^21^. For NAAA activity, 30 μg of recombinant rNAAA proteins were pre-incubated with F96 or vehicle (1% DMSO) at 37 °C for 10 min, and then 0.2 mL assay buffer (50 mM phosphate buffer, 0.1% Triton X-100, 3 mM DTT, pH 5.0) containing 25 μM heptadecenoylethanolamide (Avanti Lipids, Alabaster, AL) as substrate was added to co-incubate for further 30 min. For FAAH activity, 30 μg of FAAH proteins were incubated at 37 °C for 30 min in Tris buffer (50 mM, pH 8.0) containing fatty acid-free BSA (0.05%) and 25 μM anandamide (Sigma-Aldrich, Shanghai, China) as substrate. For Monoacylglycerol lipase (MAGL) activity, 10 μg of proteins derived from MAGL-overexpressing HEK293 cells were incubated in assay buffer (50 mM Tris, 0.05% fatty acid-free BSA, pH 8.0) with substrate (25 μM 2-oleoylglycerol). Acid ceramidase (AC)activity was measured by incubating 25 μg of proteins which were derived from AC-overexpressing HEK293 cells, with 100 μM N-lauroyl ceramide as substrate in 100 mM sodium phosphate buffer (0.1% Nonidet P-40, 150 mM NaCl, and 3 mM DTT, pH4.5) for 30 min at 37 °C. The above reactions were stopped by adding 200 μL methanol containing 1 nmol heptadecanoic acid as internal standard.

### Lipid extraction and analysis

Lipids were extracted from related tissue samples using the modified methods as mentioned^35^. Briefly, tissues (20–30 mg) were homogenized by ultrasonication in methanol/water mixture (v/v, 1:1, 1 mL) containing 100 pmoL of heptadecenoylethanolamide as internal standard. The homogenate was extracted with 4 mL chloroform and then vortex for 1 min. Lipid layer was separated by centrifugation at 3000 × *g* for 10 min and then transferred to a clean 10 mL V-bottom glass tube and dried under nitrogen (N_2_) flow. 1 mL chloroform was added to resolve dried spots and solid-phase extraction was eluted by methanol/chloroform (v/v, 1/9). The elution containing FAEs was dried under N_2_, and reconstituted in 100 μL methanol for HPLC-MS/MS analysis.

An ABI 3200 Q-Trap mass spectrometer (Applied Biosystems, USA) equipped with 1100-LC system (Agilent, Shanghai, China) was operated during this experiment. The precursor/product ion transitions in multiple reaction monitoring mode (MRM) were used for mass analysis and quantitation. The molecular ions were monitored at *m/z* 300.2/62.0 for PEA, *m/z* 326.1/62.0 for OEA, *m/z* 348.00/62.00 for AEA, *m/z* 379.10/287.10 for 2-AG, and *m/z* 313.1/62.0 for C17:1 FAE.

### 12-*O*-Tetradecanoylphorbol-13-acetate (TPA) induced ear edema in mice

Male C57BL/6J mice, weighting 20–22 g, were provided with food and water ad libitum. TPA (0.03% in acetone, 10 μL) was applied evenly to both sides of the left ears, whereas acetone with the same volume was applied to the matched right ears as control. Simultaneously, F96 (3, 10, 30 mg/kg, dissolved in 5% polyethylene glycol 400 and 5% Tween-80 in saline) was administrated by intraperitoneal injection. Animals were sacrificed after 3 h, and the ears were excised and punched in the same size (diameter = 9 mm). Ear tissues were weighed to assess edema and then divided into 2 parts immediately. One part was immersed in 5% paraformaldehyde solution for H&E assay, and the other part was weighted to quantify tissue FAE levels by HPLC-MS/MS. 129 s mice (+/+) and PPAR-α knockout mice (−/−) (20–22 g) were treated by the same procedure.

### H&E assay

Fresh mouse ear tissues were fixed in paraformaldehyde at 4 °C, and then embedded in paraffin. 5 μm-thick tissue sections were cut by Sliding microtome Leica SM2010 R (Shanghai, China), and were stained with hematoxylin and eosin (H&E) after deparaffinized with xylene. Imaging of stained areas was performed with a light microscopy (Nikon, Shanghai, China).

### Acetic acid-induced writhing in mice

Male Kunming mice, weighting 20–22 g, were provided with food and water ad libitum. F96 (10 mg/kg, i.p.) was administered 30 min before the tests. NSAID indomethacin (10 mg/kg, dissolved in 5% polyethylene glycol 400 and 5% Tween-80 in saline) was intraperitoneal injected as a positive control. CB_1_ antagonist Rimonabant (1 mg/kg, dissolved in 5% polyethylene glycol 400 and 5% Tween-80 in saline), CB_2_ antagonist SR144528 (1 mg/kg, dissolved in 5% polyethylene glycol 400 and 5% Tween-80 in saline) and PPAR-α antagonist MK886 (2 mg/kg, dissolved in 5% polyethylene glycol 400 and 5% Tween-80 in saline) were respectively injected into mice abdominal cavity at the same time with F96. After 5 min’s stimulation by acetic acid (0.6% in saline, 10 mL/kg, i.p.), the number of writhing (stretch) responses was counted for the next 20 min. 129 s mice (+/+) and PPAR-α knockout mice (−/−) (20–22 g) were treated by the same procedure.

### Spared nerve injury induced allodia in mice

The experiments were performed according to the previous reports^36^. Simply, male C57BL/6J mice were anesthetized with pentobarbital sodium. The lateral surface of the left thigh proximal to the knee was longitudinally incised to expose the left sciatic nerve and separate the three terminal branches: sural nerve, common peroneal nerve and tibial nerve under a stereomicroscope. The common peroneal and tibial nerves were tightly ligated with suture (6-0 suture), and then were cut by a micro scissor. The wound was sutured, cleaned with 75% ethanol, and covered with sterilized gauze pads. Sham controls involved exposure of the sciatic nerve without any branch lesion. F96, Rimonabant, SR144528 and MK886 were administrated as described in 2.8 at the 3rd and 7th day after surgery. Gabapentin (40 mg/kg, i.p.) was intraperitoneally injected as a positive control. Withdraw threshold was assessed using the automated Dynamic Plantar Aesthesiometer system (Ugo Basile, Comerio, Italy).

### Open field assay

Male ICR mouse (20–22 g) was placed in the centre of the open field apparatus (length 20 × width 20 × height 30 cm) after F96 treatment (30, 100, 300 mg/kg, i.p.) for 1 h, 3 h and 6 h. Data of mouse motion trails during the first 300 sec of each time period were analyzed. The total distance and centre distance moved in the arena, and the time spent in the center area were monitored by ZH-ZFT type inner open field test system (Huaibei Zhenghua Company, China).

### Zebrafish assay

Zebrafish embryos were used to assess acute toxicity of F96. All tests were performed in triplicate and each set consisted of 10 developing embryos initially. The embryos undergoing early cleavage (two/four celled stages) or 24 h post-fertilization were exposed to different concentrations (0.1, 0.3, 1, 3, 10 mg/L) of F96 or ibuprofen, a commercial NSAID. Compounds were separately dissolved in dimethyl sulfoxide (DMSO) to make a 10 mM stock solution. The treatment solutions for toxicity tests were obtained by the dilution of the stock solutions with embryo medium. Corresponding zebrafish medium and carrier controls (0.1% DMSO) were maintained. All embryos were put in 96-well plates (one egg per hole) in which the test solutions were renewed every 24 h, and cultured at 28.5 °C. Observations were made hourly during the early embryogenesis, thereafter, once every 24 h and up to total 72 h. Dead embryos were moved once detected. Morphologies of embryos/larvae were carefully observed with an inverted microscope and images were captured using an image analyzer. The larvae hatching rate, total mortality, average heartbeats, and body length of all embryos/larvae was recorded on 72 h.

### Data analyses and statistics

All statistical analyses were completed by using GraphPad Prism version 5.01. Data were showed as the mean ± SEM and were evaluated by one-way analysis of variance (ANOVA) followed by Dunnett’s test for multiple comparisons. *P *< 0.05 was considered statistically significant.

## Additional Information

**How to cite this article**: Yang, L. *et al.* Potential analgesic effects of a novel *N*-acylethanolamine acid amidase inhibitor F96 through PPAR-α. *Sci. Rep.* **5**, 13565; doi: 10.1038/srep13565 (2015).

## Figures and Tables

**Table 1:** Effects of F96 on enzyme activities.

	FAAH activity	MGL activity	AC activity	NAAA activity
Vehicle	100 ± 3.18	100 ± 8.22	100 ± 12.31	100 ± 3.57
F96 (10 μM)	80.46 ± 2.74	90.07 ± 3.22	96.73 ± 8.01	5.09 ± 0.30

Values present as mean ± S.E.M.
